# The Thymus in Chagas Disease: Molecular Interactions Involved in Abnormal T-Cell Migration and Differentiation

**DOI:** 10.3389/fimmu.2020.01838

**Published:** 2020-09-02

**Authors:** Ana Rosa Pérez, Juliana de Meis, Maria Cecilia Rodriguez-Galan, Wilson Savino

**Affiliations:** ^1^Instituto de Inmunología Clínica y Experimental de Rosario, CONICET-Universidad Nacional de Rosario, Rosario, Argentina; ^2^Centro de Investigación y Producción de Reactivos Biológicos, Facultad de Ciencias Médicas, Universidad Nacional de Rosario, Rosario, Argentina; ^3^Laboratory on Thymus Research, Oswaldo Cruz Institute, Oswaldo Cruz Foundation, Rio de Janeiro, Brazil; ^4^National Institute of Science and Technology on Neuroimmunomodulation, Oswaldo Cruz Institute, Oswaldo Cruz Foundation, Rio de Janeiro, Brazil; ^5^Rio de Janeiro Research Network on Neuroinflammation, Oswaldo Cruz Institute, Oswaldo Cruz Foundation, Rio de Janeiro, Brazil; ^6^Inmunología, CIBICI CONICET, Facultad de Ciencias Químicas, Universidad Nacional de Córdoba, Córdoba, Argentina

**Keywords:** Chagas disease, thymocyte depletion, thymic epithelial cells, gene expression, cell adhesion and migration

## Abstract

Chagas disease, caused by the protozoan parasite *T. cruzi*, is a prevalent parasitic disease in Latin America. Presently, it is spreading around the world by human migration, thus representing a new global health issue. Chronically infected individuals reveal a dissimilar disease progression: while nearly 60% remain without apparent disease for life, 30% develop life-threatening pathologies, such as chronic chagasic cardiomyopathy (CCC) or megaviscerae. Inflammation driven by parasite persistence seems to be involved in the pathophysiology of the disease. However, there is also evidence of the occurrence of autoimmune events, mainly caused by molecular mimicry and bystander activation. In experimental models of disease, is well-established that *T. cruzi* infects the thymus and causes locally profound structural and functional alterations. The hallmark is a massive loss of CD4^+^CD8^+^ double positive (DP) thymocytes, mainly triggered by increased levels of glucocorticoids, although other mechanisms seem to act simultaneously. Thymic epithelial cells (TEC) exhibited an increase in extracellular matrix deposition, which are related to thymocyte migratory alterations. Moreover, medullary TEC showed a decreased expression of AIRE and altered expression of microRNAs, which might be linked to a disrupted negative selection of the T-cell repertoire. Also, almost all stages of thymocyte development are altered, including an abnormal output of CD4^−^CD8^−^ double negative (DN) and DP immature and mature cells, many of them carrying prohibited TCR-Vβ segments. Evidence has shown that DN and DP cells with an activated phenotype can be tracked in the blood of humans with chronic Chagas disease and also in the secondary lymphoid organs and heart of infected mice, raising new questions about the relevance of these populations in the pathogenesis of Chagas disease and their possible link with thymic alterations and an immunoendocrine imbalance. Here, we discuss diverse molecular mechanisms underlying thymic abnormalities occurring during *T. cruzi* infection and their link with CCC, which may contribute to the design of innovative strategies to control Chagas disease pathology.

## Introduction

Chagas disease is caused by the hemoflagellate protozoan *Trypanosoma cruzi*. Infection was initially an enzooty maintained among wild animals and *Reduviidae* family insects as vectors. The classical vectorial pathway occurs by contact with feces or urine of hematophagous triatomine bugs, which are frequent in Latin American endemic areas (1, 2). After the triatomine bite feed with blood, it usually defecates close to the bite. The parasites present in the feces then enter through the damaged skin when the person scratches the itchy bite or, through mucous membranes like ocular conjunctiva. Particularly, mucosal oral transmission has been associated with high mortality and morbidity, increased prevalence, and severity of the cardiac pathology (3–7). Moreover, parasites can be transmitted by contaminated blood transfusion, organ transplantation, and vertically. These latter types of transmission are also responsible for Chagas disease dissemination in non-endemic areas, including the USA, Europe, and Asia (8, 9). Nearly 6–7 million people in Latin America plus 1 million in the USA are infected with *T. cruzi* with 670.000 premature disability and death per year worldwide (8–10).

Human Chagas disease shows a short acute phase (2 months), a period in which parasites are numerous in blood and tissues. During this phase, *T. cruzi* can infect host skeletal muscle, heart, lymphoid cells, adipocytes, mucosal sites, neurons, glands, liver, among others. Moreover, in some target tissues, damage can persist in the chronic phase of the disease (3, 11–13). Following the acute phase, patients enter into a long latent phase, with no symptoms and scarce parasitism, which can remain silent for the rest of life. After 10–30 years, one-third of infected patients eventually develop clinical symptoms as CCC, megacolon, or megaesophagus (14). The CCC is associated with mononuclear cell infiltrate, fiber damage, fibrosis, and rare presence of parasites. The inflammatory infiltrate in CCC exhibits more CD8^+^ over CD4^+^ T cells and hearts from patients present high granzyme A expression, suggestive of cytotoxicity in the tissue (15–19).

## The Thymus in Chagas Disease

Since Chagas disease was described in 1909, numerous studies have been conducted on the pathogenesis of the disease and the evolution of both acute and chronic phases of infection (1, 2). However, dissection of diverse pathogenic mechanisms remains open to investigation. Upon recognition that *T. cruzi* persists in the host during the chronic phase, the hypothesis stating that the chronic tissue damage is mediated and maintained by inflammatory reactions caused by the continuous parasite's cycles of replication was reinforced (20) and the autoimmune hypothesis of the disease (the most accepted until then) was questioned (21). However, there is profuse evidence on the occurrence of autoimmune events, mainly caused by molecular mimicry and bystander activation (22). These mechanisms are not mutually exclusive, and both likely operate conjointly. In any case, it is well-established that *T. cruzi* infects the thymus and causes locally structural and functional alterations (23). Therefore, understanding the possible implications of thymic changes in the immunopathology of this parasite infection may help to appreciate new edges of the disease.

Studies in animal models of acute Chagas disease revealed marked thymus atrophy, mainly caused by thymocyte death, as well as functional alterations, including an abnormal output of immature and mature cells (24). These data suggested that both systemic and thymic inflammation might drive to central tolerance defects, while simultaneously increase the suspicion of a thymic involvement in the development of CCC, although this issue remains uncertain. In this sense, the following questions still need to be approached:

- Can the observations made in the thymus of *T. cruzi* acutely infected mice be transposed to what happens in humans?
- Can thymic alterations persist during the chronic phase?
- Is the thymic atrophy a mere side effect secondary to an immunoendocrine imbalance?
- Which are the consequences of cellular and molecular alterations promoted by the intrathymic parasite infection?
- Which of these thymic abnormalities may contribute to CCC?

Nevertheless, studying the human thymus in the context of Chagas disease to answer these questions is not an easy task. In humans, determining the occurrence of atrophy requires non-invasive techniques, which also prevents obtaining tissue biopsies for cytometry, molecular, or microscopic studies. Thymus size can be ascertained in children at an early age by ultrasound but requires qualified operators. In adults, there is normal age-associated atrophy together with a huge increase in adipose tissue, making it still much more difficult to evaluate the organ.

Additionally, the acute phase of vector-induced infection usually remains unnoticed, which prevents thymic evaluation as it occurs. Moreover, infection mainly occurs in rural areas where it is unlikely to have adequate medical equipment and suitable personnel. Congenital cases, when detected, are rapidly treated, preventing the tracking of putative changes in the organ size. Future non-invasive studies, including determination of T-cell excision circles, which reflect thymocyte export to the periphery, will hopefully be carried out in orally-infected symptomatic individuals, and provide clues to unravel important thymus-related issues in both children and adults undergoing Chagas disease.

Beyond all these relevant points, which remain to be unveiled, there is no doubt that in the murine model of Chagas disease profound thymic alterations occur, which may be of relevance in terms of both thymic selection and immune competition. Accordingly, we discuss below the most relevant cellular, molecular, and functional alterations observed in the thymus of infected mice and discuss their possible resemblance to the human disease.

## The Thymic Microenvironment in *T. cruzi* Infected Mice

During a variety of systemic infections, several changes occur in the thymus leading to the local presence of both soluble factors, cytokines, chemokines and pathogen-derived antigens that could alter not only the thymic microenvironment but also the normal T cell differentiation process, including the export of mature T lymphocyte to secondary lymphoid organs (the normal process of differentiation is summarized in Box 1).

Box 1 **Intrathymic T-cell development.**

In physiological conditions, several T cell lineages arise in the thymus including conventional αβT cells, γδT cells, CD4^+^FoxP3^+^ regulatory T cells (tTreg), and NKT cells. Recently, more lineages have been added to the list, including several subtypes of innate T cells (25, 26).

From the arrival of the bone marrow-derived T-cell precursors into thymus until the ultimate export of mature T cells to the periphery, a migratory journey inside the thymus takes place, involving a large number of interactions which promote the complex process of T-cell differentiation. This process necessarily depends on T-cell receptor (TCR) gene rearrangement and on the interaction of TCR with self-peptides presented by class I or class II proteins of the major histocompatibility complex (MHC) expressed by microenvironmental cells. Nevertheless, other types of interactions are also relevant including those mediated by chemokines, cytokines, lectins, lipid signalling molecules, hormones and extracellular matrix (ECM) proteins (27–30). Once bone marrow-derived T-cell precursors enter the thymus through blood vessels located at the cortico-medullary junction, they differentiate in DN thymocytes. Based on the expression of CD44 and CD25, the sequential maturation process of DN cells indicates that DN1 thymocytes develop to DN2 and DN3 thymocytes, which migrate to the subcapsular area of the thymic lobules, where they rearrange TCR-β chain-encoding-genes, express the pre-TCR receptor and proliferate. At the DN3 stage, CXCL12/CXCR4-mediated interactions contribute to cell proliferation and differentiation toward the DN4 and subsequently to the DP stage (28, 31, 32). Thymocytes that do not experience a productive TCR gene rearrangement during the DN stage die by apoptosis, whereas those expressing productive TCRs evolve to DP cells, which in turn can recognize self-peptides in the context of MHC molecules. This interaction determines the events of negative and positive selection (28, 33, 34). The intrathymic negative selection process is essential to establish self-tolerance in the T-cell repertoire, deleting thymocytes exhibiting TCRs with high-avidity for self-peptides (33). Most of the DP thymocytes die due to lack of positive selection as their TCR cannot recognize own MHC-peptide complexes on thymic microenvironmental cells (*death by neglect*) possibly through induction of apoptosis mediated by endogenous steroids (35, 36). Positively selected thymocytes become committed to CD4^+^ or CD8^+^ T lineages, depending on the class of MHC molecule with which the TCR interacts. Particularly, physiological levels of glucocorticoids (GC) have an important role in the normal thymocyte development, regulating essential process like antigen-specific selection, TCR-dependent activation, ultimately shaping the T cell repertoire [revised in (37, 38)].

Besides, among CD4^+^ T-cells, two distinct groups of cells with opposite roles differentiate within the thymus: the classical CD4^+^ T helper lymphocytes and tTregs (28, 39). Moreover, innate memory CD8^+^ T cells (T_IM_-CD8^+^) are generated as a lineage different from conventional CD8^+^ T cells. Some reports also demonstrated that thymic selection of T_IM_-CD8^+^ cells results from the interaction with hematopoietic cells present in the thymus and not with TEC, as is the case of conventional T cells (25, 26, 40, 41). As an overall result of these events, a vast repertoire of T cells able to react with peptides restricted by MHC molecules is generated.

Once the differentiation process is complete, mature single positive (SP) cells congregate near medulla vessels and are exported to the periphery. These recently exported cells are known as recent thymic emigrants (RTE). They are naïve T lymphocytes that migrate mainly toward the T-cell-dependent areas of secondary lymphoid organs.

A first key point regarding the thymus in experimental Chagas disease is the fact the *T. cruzi* can invade the organ. The parasite can infect thymic microenvironmental cells, including phagocytes and TEC (42–44). Medullary TEC from infected mice exhibited a significant decrease in AIRE gene expression (45), which might be related to disruption in the negative selection of the T-cell repertoire. Nevertheless, the levels of AIRE expression remain rather controversial since no differences were also found in another study (46). Although definitive evidence is lacking, differences reported may be due to the fact that in one experiment (45), measurements were done taking separated TEC, whereas in the other, the whole thymus was used as the primary source of RNA (46). Previous studies showed that GC treatment in mice transiently reduced the number of AIRE^+^mTEC (47). Thus, it is conceivable that the diminution in AIRE^+^TEC numbers during infection may be secondary to the rise in systemic glucocorticoids (GC) levels.

*In vitro*, the parasite invades growing TEC, modifying distinct biological features. These changes include a decrease proliferation, enhanced production of extracellular matrix proteins (ECM), and the corresponding receptor expression, leading to the consequent enhancement in the ability of TEC to adhere developing thymocytes, particularly on those cells infected by the parasite (48) (Table 1).

**Table 1:** *Trypanosoma cruzi* infects mouse thymic epithelial cells (TECs)^*^, affecting TEC replication and adhesion of thymocytes.

	**Non-infected**	**Infected**	***p*-value**
Thymic epitelial cell numbers^**^			
Total TEC numbers	7.00 ± 0.63	3.20 ± 0.33	*p* < 0.02
BrdU^+^ TEC numbers	56 ± 1.10	14 ± 0.40	*p* < 0.02
Association index applied to quantify thymocyte adhesion to TECs^***^	33.6 ± 2.5	80.0 ± 4.4	*p* < 0.05
	**TEC/Tc(–)**	**TEC/Tc(+)**	
	67 ± 0.9	200 ± 21.5	*p* < 0.05

* *The TEC line IT-76M1 was originally developed from a primary culture of BALB/c thymic stromal cells and was kindly provided by Dr. T. Itoh (Tohuku University Medical School, Sendai, Japan). TEC cultures were allowed to adhere to culture flasks (1 × 10^5^ cells) and were infected with T. cruzi 24 h later, being then washed extensively. Trypomastigote forms of the Colombian strain were applied to infect TEC cultures. After 6 h, free parasites were discarded by repeated washings, and the TEC cultures were maintained for further 48 h*.

** *The numbers of cultured TECs were ascertained by directly counting single-cell suspensions after detaching the cells Additionally, the proliferative status of the infected and uninfected TEC cultures was analyzed using a BrdU incorporation assay (30 min at 37°C). The presence and counting of BrdU^+^ cells were determined by fluorescence microscopy*.

*** *Infected or non-infected TEC cultures were maintained for 48 h, being then incubated with 5 × 10^6^ thymocytes, obtained by the mechanical disruption of control thymuses, in serum-free RPMI medium for 30 min at 37°C. Floating, non-adherent thymocytes were removed and the plates were fixed in methanol and stained with Giemsa solution. The number of adhered thymocytes per at least 1,000 TECs were directly counted, and the association index (AI) was calculated as follows*:

AI, TECs with bound thymocytes × thymocytes bound to TECs × 100

total TEC number total TEC number

*All data showed in this table were retrieved from Farias-de-Oliveira et al. (48)*.

When studying the expression of microRNAs in TECs from infected animals, many of these non-coding RNAs were up- or down-regulated. The bioinformatic-based simulation revealed that various down-regulated miRNAs can result in the up-regulation of biological processes involving ECM proteins, cell adhesion, and cell migration (49). In keeping with these findings, the expression of cell adhesion and cell migration-related molecules, including chemokines as well as ECM proteins by TEC is enhanced in thymuses from infected mice (23, 42, 50, 51), as well as after *in vitro* infection (48) with functional consequences as further discussed in detail below.

## Thymocyte Depletion in Acute *Trypanosoma cruzi* Infection

Thymic atrophy seems to be a common finding in various experimental infections; being however much less documented in humans (52). As mentioned before, DP thymocyte loss by apoptosis is evident during the acute phase of *T. cruzi* infection (12, 24, 42, 53, 54), and diverse molecules have been raised as candidates to enhance thymocyte death (55, 56). However, the most relevant way involved in *T. cruzi*-induced apoptosis of developing DP thymocytes seems to be related to the systemic rise of GC levels as a consequence of the host's stress response to the infection (57, 58). Regarding the *T. cruzi* infection, it has been clearly proved that complete GC ablation by adrenalectomy or the blockade of the GC receptor (GR) by RU486 administration clearly prevented DP cell loss by apoptosis (56, 57, 59).

Two main apoptotic pathways have been described: one of them is the extrinsic pathway, dependent on membrane “death receptors” and caspase 8, while the other is the intrinsic or mitochondrial pathway, dependent on Apaf-1 and caspase 9. Both pathways lead to the activation of caspase 3, which executes the programmed cell death. There is considerable evidence that in diverse cell types, including thymocytes, GC cause apoptosis via the intrinsic pathway (60–62). These pathways were regulated by proteins from the Bcl-2 family consisting of pro- and anti-apoptotic proteins, like Bax, Bid, Bim, and Puma (61). Also, in thymocytes, GC can modulate the balance between members of this family, triggering the release of cytochrome c by mitochondria (60, 63, 64). Cytochrome c participates in the formation of the apoptosome with Apaf-1 and pro-caspase9, leading to caspase 9 activation with consequent activation of caspase3. These data are reinforcing by the fact that, in thymocytes, GC-driven apoptosis is prevented in mice deficient in the pro-apoptotic proteins Bak and Bax (65), Apaf-1 (63, 66) or caspase 9 (67).

Despite the fact that the exact apoptotic program initiated by GC has not been fully described in DP thymocytes, the use of specific peptide inhibitors has implicated caspases 8, 9, and 3 in this process during *T. cruzi* infection (68). These findings suggest that both intrinsic and extrinsic apoptotic pathways may be involved. However, studies performed in C57BL/6 mice deficient in both TNF-receptors (TNF-R_1/2_ double knockout mice) showed that the genetic ablation of TNF-triggered death pathways failed in preventing *T. cruzi*-induced cortical thymocyte depletion (56). Other series of studies discarded the involvement of interactions mediated by Fas/Fas-L or even perforin in triggering cell death within the thymus (55). These data strongly indicate that apoptotic pathways activated through death receptors are not involved in the *T. cruzi*-driven thymic atrophy. Yet, how can be explained the activation of caspase 8, observed by Farias de Oliveira and co-workers? (68). It is possible that caspase 9, in addition to cleaving pro-caspase 3, also activates lysosomal cathepsin B, which in turn activates caspase 8 providing an alternative route for caspase 3 through the mitochondrial pathway (64). This hypothesis remains to be tested since the release of cathepsin B has not been yet evaluated during infection with *T. cruzi* in DP thymocytes. Moreover, some findings suggest that GC can act, in addition to caspase 9, through caspase 8 (52, 58, 59, 64, 69–71). In any case, the findings summarized above strongly suggest that the mitochondrial pathway of apoptosis is triggered in DP thymocytes because of the GC rise driven by pro-inflammatory cytokines (56–58).

Other regulatory mechanisms may also influence the outcome of the GC-induced thymus atrophy in *T. cruzi* infected animals. It is known that the cellular response to GC is regulated by the availability of different GR isoforms. The GRα and GRβ receptors are the most abundant (72). GRα binds GC, while GRβ does not bind the hormone or transactivate target genes, acting as an inhibitor of GRα activity (72). Interestingly, *T. cruzi*-infected thymuses progressively down-regulate GRα gene expression (73). Although GRβ thymic contents have not been evaluated, this process may be a compensatory mechanism conferring thymocytes greater resistance to the deleterious effects of the hormone during the acute infection in mice (73). Moreover, tissue GC availability can also be modulated by 11β-hydroxysteroid dehydrogenases (11β-HSD)-1 and 2. While 11β-HSD1 increases the availability of active GC, 11β-HSD2 converts active GC to a less active metabolite (72). Interestingly, thymuses from *T. cruzi* infected animals, show an enhancement of 11β-HSD1 gene expression, paralleled by increased production of functional GC by TEC, revealing an active intrathymic steroidogenic machinery (73), which synergizes the local consequences of systemic GC (74, 75).

Additionally, it has been shown that prolactin (PRL), which is enhanced intrathymically during *T. cruzi* infection, counteracts the deleterious effects of GC. Accordingly, the administration of metoclopramide (a PRL secretagogue), significantly prevented the thymic atrophy as well as the abnormal exit of DP thymocytes (73). Interestingly, the onset of thymic atrophy in *T. cruzi*-infected animals is associated with an inverse balance between the intrathymic expression of GR and PRL receptor (PRLR), possibly altering the sensitivity of DP thymocytes to GC-induced apoptosis (73). Mature CD4^+^ and CD8^+^ SP thymocytes are much more resistant to the GC proapoptotic effects than the immature DP cells (76), and this fact is clearly seen during the infection, by the increase in the intrathymic relative numbers of SP thymocytes.

Other molecules possibly involved in thymic atrophy during *T. cruzi* infection have been described, although much less studied than GC. Yet, it cannot be ruled out that they might synergize their effects. Galectin(Gal)-3 is a carbohydrate-binding protein that modulates cell death in mature and immature T cells (77). Gal-3 has a sequence in the protein core similar to Bcl-2, a well-characterized suppressor of apoptosis. Intracellularly, Gal-3, like Bcl-2, maintains mitochondrial integrity and prevents cytochrome c release, blocking cell death (77, 78). Nevertheless, in contrast to the antiapoptotic function of intracellular Gal-3, extracellular Gal-3 can directly induce thymocyte death (12, 77). In the normal murine thymus, Gal-3 is mainly restricted to the medulla, where it is produced and accumulates on the cell surface of TECs and phagocytic cells (79). Interestingly, in the thymus of *T. cruzi* infected mice, protein contents of Gal-3 are increased in the medulla, and to a lesser extent in the cortex (12). Additionally, thymocytes from infected mice showed an enhanced Gal-3 gene expression and increased cytoplasmatic protein storage (12), suggesting that the accumulation of Gal-3 may be associated with the depletion of DP thymocytes. Moreover, thymuses from Gal-3 knockout mice showed diminished cellularity than wild type mice, but the proportions of thymocyte subsets are maintained (12, 80). When infected, and despite the low thymic cellularity exhibited by Gal-3 knockout mice, DP thymocyte proportions were preserved as compared to wild-type mice (12, 80). Therefore, understanding the exact role of Gal-3, as well as other pro-apoptotic family members like Gal-1, in thymic atrophy during *T. cruzi* infection requires further studies.

It has been shown that extracellular ATP, acting via P2X7 receptors, increases DP thymocyte sensitivity to death by rising membrane permeability during the peak of *T. cruzi* acute infection (81, 82). Deprivation of intrathymic key cytokines may be also involved in the loss of thymocytes. In this regard, previous studies showed that thymocyte responses to mitogens in *T. cruzi* acutely infected mice are decreased due to compromised IL-2 secretion and high levels of IL-10 and interferon IFN-γ (24).

Lastly, some studies showed that thymic atrophy was not induced by a non-virulent strain of *T. cruzi*, suggesting that parasite-derived virulence factors may be involved in the death of thymocytes. In keeping with this hypothesis, some studies indicate that the parasite-derived enzyme trans-sialidase may be involved in the thymic atrophy (83, 84). Despite that *T. cruzi* can infect the thymus (43, 46, 58) and release locally antigens, some studies have shown that when trans-sialidase is artificially shed to circulation, it induces thymocyte apoptosis (85). Moreover, mice treated with trans-sialidase displayed enhanced thymocyte apoptosis within the thymic nurse cell complexes, findings resembling thymic alterations in infected animals (83). Additionally, the trans-sialidase treatment *per se* was able to promote a decrease of thymocyte proliferative ratios after Concanavalin A stimulation, similar to those observed in the experimental models of *T. cruzi* infection. Interestingly, the administration of trans-sialidase neutralizing antibodies in *T. cruzi* infected mice significantly prevented thymocyte apoptosis (83). Furthermore, lactitol, a competitive inhibitor of trans-sialidase that blocks the transfer of sialyl residues by the enzyme, was able to prevent thymocyte depletion (86, 87). Strikingly, when acute infection (and the conceivable shedding of trans-sialidase) is controlled by benznidazole administration, thymus atrophy is not so evident (88).

Trans-sialidase probably acts upon CD43 (89). Nevertheless, the molecular mechanisms involved in CD43-mediated apoptosis of DP thymocytes during *T. cruzi* infection deserve a more profound investigation. However, beyond the probable direct effects caused by an active trans-sialidase on DP thymocytes, it is also conceivable that the immune response triggered against this molecule activates the HPA axis and/or the intrathymic machinery of GC synthesis, causing GC rise with consequent death of DP thymocytes. Yet, the existence of these circuitries remains to be determined.

**Figure 1:**
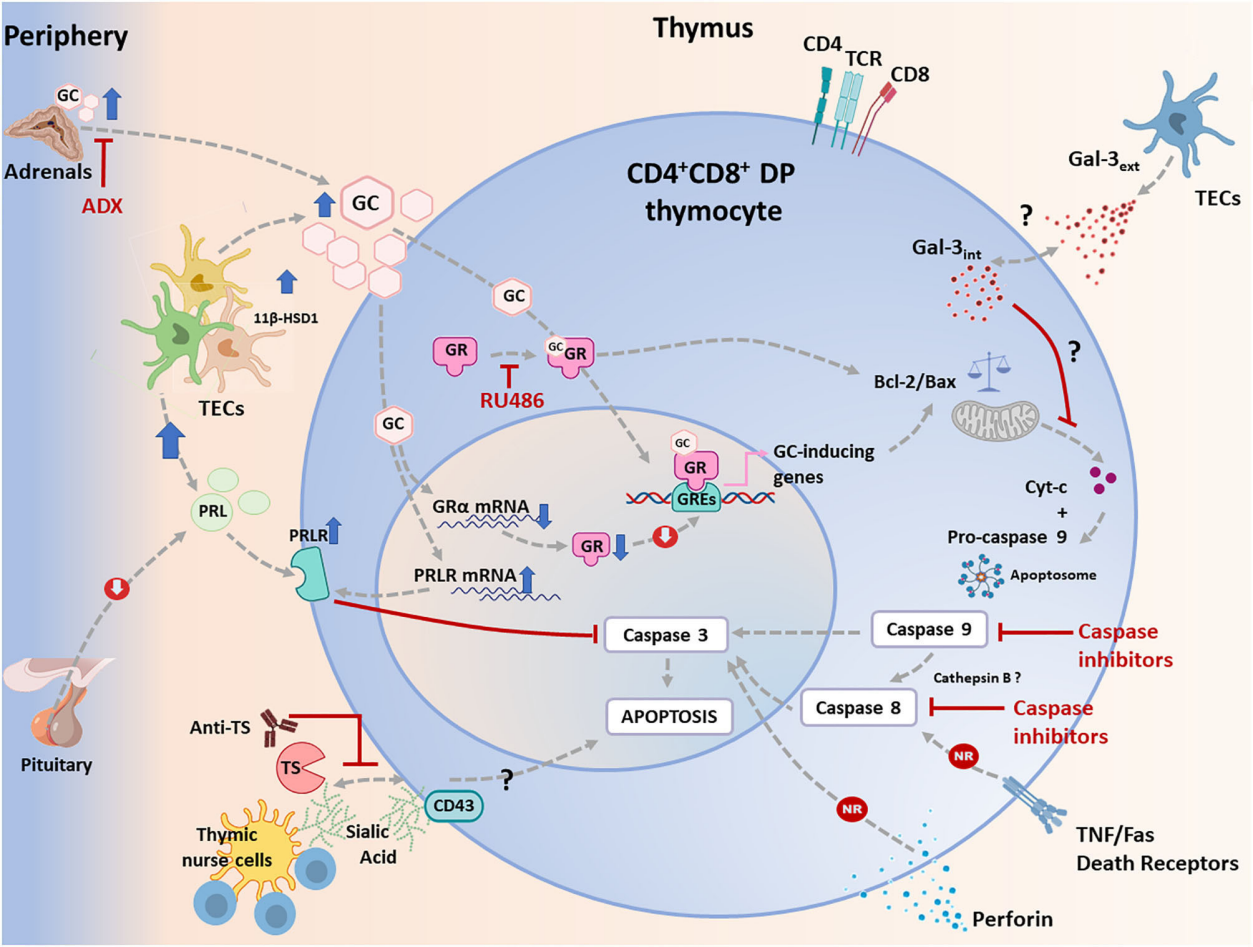
Apoptotic signaling pathways triggered in CD4^+^CD8^+^ double positive (DP) thymocytes during *T. cruzi* infection in mice. Apoptosis can be initiated via two different routes including extrinsic and intrinsic pathways, which converge in a caspase activation cascade. The most relevant physiological way involved in *T. cruzi*-induced apoptosis of developing CD4^+^CD8^+^ DP thymocytes is linked to the systemic rise of glucocorticoid (GC) levels secondary to the host's stress response to the infection. This process is synergized by an increase in intrathymic production of GC by thymic epithelial cells (TECs), conjointly with an increased availability of induced-11 b-hydroxysteroid dehydrogenase(11β-HSD)-1. As proved, GC-driven apoptosis is greatly prevented by adrenalectomy or by the blockade of GC receptor (GR) with RU486. In addition, GC can stimulate the intrinsic pathway either genomically or non-genomically, a process that involves proteins of the Bcl-2 family. Pro-apoptotic and anti-apoptotic proteins of the Bcl-2 family are up-and down-regulated respectively, leading to the release of cytochrome c, which then forms the apoptosome together with pro-caspase 9 and Apaf-1, leading to the activation of caspase 9. This enzyme then activates caspase 3. During *T. cruzi* infection, cytochrome c is enhanced, and caspase 9 is activated in CD4^+^CD8^+^ DP thymocytes. As shown, inhibition of caspase 9 weakens CD4^+^CD8^+^ thymocyte death. In parallel, increased systemic and intrathymic GC production leads to a diminution of GR expression, probably as a negative regulatory loop tending to control unnecessary harmful effects upon the gland. Moreover, *T. cruzi* infection induces the synthesis of prolactin (PRL) by TECs, while GC improved the expression of the PRL receptor (PRLR) in TECs. Increased PRLR signaling then counterbalances GC-induced effects, preventing caspase 3 activation. Extrinsic signals can be initiated by cell death ligands (FasL and TNF-α) and involve caspase 8, but these routes seem to be non-relevant (NR) during *T. cruzi* infection, since atrophy takes also place in TNFR_1+2_ double knock-out mice or Fas knock-out animals. Similar results were observed in perforin knock-out animals. Since caspase 8 is also active, a possible alternative route for its activation may involve cathepsin B, but this route deserves confirmation. Yet, it is clear that caspase 8 inhibition reduces thymocyte death during *T. cruzi* infection. Thymocyte apoptosis can also be triggered by trans-sialidase (TS), a *T. cruzi* derived enzyme, and this effect is partially blocked by anti-TS antibodies against. Trans-sialidase transfers sialic acid residues between the parasite and thymocytes, and probably acts through CD43. The subsequent steps leading to apoptosis are unknown. Other pathways described, as caused by intracellular and extracellular Gal-3, seems to influence the intrinsic apoptotic pathway.

Conjointly, these data indicate that thymic depletion of DP thymocytes in acute *T. cruzi* infection is primarily caused by GC enhancement, and perhaps this pathway can be modulated by multiple interactions involving both endogenous factors and infectious agent-derived moieties. This complex scenario is summarized in Figure 1.

## Other Mechanisms That Compromise Thymic Cellularity in the Context of *T. cruzi* Infection

It is well-established that, during the process of thymic atrophy, the loss of DP thymocytes is followed by a drop in absolute numbers of thymic CD4^+^FoxP3^+^ tTreg cells, as well as CD4^+^ and CD8^+^ SP thymocyte (90). Nonetheless, *T. cruzi* infection induces an increase in the proportion of tTreg cells within the thymic CD4^+^ SP T cell compartment, apparently as result of an increase in its self-renewal capacity compared to CD4^+^FoxP3^−^ T cells (90). These findings are independent of the mouse or parasite genetic strain, but can vary in intensity (90–92).

Interestingly, some findings demonstrate a negative regulatory role for CD28 in inhibiting differentiation of SP thymocytes, possibly declining thymic selection (93). In contrast, more recent findings suggest that CD28-mediated transduction signals favor CD4^+^FoxP3^+^CD25^+^ tTreg development and proliferation in steady-state conditions (94, 95). Therefore, it is possible to presume that given the inflammatory environment that is established by *T. cruzi* infection, CD28-mediated co-stimulatory signals could, in part explain the relative increase of tTregs among CD4^+^SP cells. Nevertheless, the precise role of CD28 in tTreg development in the thymus during *T. cruzi* infection has not been addressed yet and deserves further investigation.

In addition, tTreg differentiation from CD4^+^CD25^+^Foxp3^−^ → tTreg appears to be highly dependent on IL-2 and IL-15 secretion by CD4^+^ SP T cells located in close contact with medullary TEC (96). Both IL-2 and IL-15 trigger signaling pathways in T-cell precursors through receptor complexes containing the common cytokine receptor γc-chain subunit, which is involved in the activation of Stat5 proteins and Foxp3 expression (97–99). Consequently, the failure of medullary CD4^+^CD25^+^Foxp3^−^ T cells to differentiate into tTreg *in T. cruzi* infected animals may be at least in part, the result of the reduced IL-2 intrathymic contents (90). Interestingly, in non-lethal models of disease, the partial thymic recovery observed after the acute phase, were linked to the increase in the numbers of IL-2R expressing cycling cells (24). Accordingly, *T. cruzi* may not only induce the depletion of cytokines that trigger the γc-chain family of receptors, but also alter the expression of these receptors, impairing the normal tTreg development (90, 100, 101), with potential consequences in the appearance of autoimmune CD4-dependent events.

In addition, the Th1 milieu that is established during *T. cruzi* infection may severely affects TCR affinity and the strength of co-stimulatory signals. Thus, the intrathymic presence of pro-inflammatory cytokines can also modulate the selection process, affecting their thresholds of survival and death (102, 103). Previous studies carried out in *T. cruzi* infected mice knock-out for TNF-R_1+2_, IFN-γ, iNOS, or IL-12, revealed that only IL-12 is likely involved in thymic atrophy by apoptosis and DP loss (104). Possibly, signals driven by this cytokine may affect the process of negative selection, but this issue has not been yet confirmed. In a second vein, IL-4 and IL-10 are not involved in thymocyte survival or death after infection, coinciding with data showing that the absence of both cytokines do not affect thymocyte cell populations in physiological contexts (102).

## The Intrathymic T-Cell Repertoire and Exit of Potentially Autoreactive Cells

The autoimmune hypothesis of Chagas disease points out to the presence of T and B autoreactive cells in the chronic phase, which may be involved in organ damage. Accordingly, it might be possible that these autoreactive cells have not completed their thymic education correctly (negative central tolerance) before their exit to the periphery. Taking in advantage the expression of endogenous superantigens (also called Minor lymphocyte stimulating -Mls- antigens) by thymic stromal cells in mice, is possible to evaluate changes in the selection process through the screening of TCR-Vβ repertoire (for more details, see Box 2) (105). Studies focused in describing the T cell repertoire in *T. cruzi* infected animals in terms of TCR-Vβs rearrangements, confirmed that enhanced numbers of T cells bearing potentially autoreactive TCRs (that in physiological conditions, should have been deleted in the thymus by negative selection) were present in the secondary lymphoid compartment (106).

Box 2 **Minor lymphocyte stimulating antigens help to evaluate positive and negative selection processes in the thymus.**

Minor lymphocyte stimulating (Mls) antigens are encoded by endogenous superantigens that were incorporated into the genome of distinct inbred mice from the mouse mammary tumor virus family (105, 107). It has been shown that Mls antigens expressed in the thymus induce depletion of T cells bearing Mls-reactive TCR-Vβ domains, allowing the exploration of the conditions for both positive and negative selection events. These studies revealed the importance of Mls and Mls-like products in the shaping of the T cell repertoire and revealed the presence in the periphery of T cell clones with “forbidden TCRs” that may have escaped from selection processes (105, 108).

Diverse mouse strains with distinct Msl phenotypes have been used to evaluate the peripheral T cell repertoire [revised in (108, 109)]. Particularly, the following mouse strains were used to evaluate the T cell repertoire after *T. cruzi* infection:

a) BALB/c mice, which contain Mls^a^ antigens, leading to the intrathymic TCR-Vβ 3,5,11,12 depletion (106, 110, 111)
b) C57BL/6 mice do not express antigens Mls antigens; therefore they do not deplete specific TCR-Vβ clones during negative selection (112).
c) C3H/HeJ mice, which contain Mls^c^ antigens, leading to the intrathymic TCR-Vβ 3,5,11,12 depletion (112).
d) CBA/J mice, which contain Mls^a^ and Mls^c^ antigens, leading to the intrathymic TCR-Vβ 3,5,6,7,8.1,9,11,12 depletion (110).

All these studies were focused on the evaluation of TCR-Vβ patterns in thymic or peripheral SP T cells, except one which also evaluated the TCR-Vβ repertoire in thymic and peripheral DP T cells (106). This particular work revealed the existence of DP T cells in the periphery that would have escaped from the thymus, bypassing the process of negative selection.

These studies were carried out in BALB/c mice, taking into the advantage that in these animals, thymocytes expressing the “prohibited” segments Vβ5 and Vβ12 are normally deleted during negative selection, whereas thymocytes bearing Vβ8 are not eliminated and are found in the periphery after maturation process. The most striking observation in *T. cruzi* infected BALB/c mice was the anomalous presence in the periphery of immature and activated CD25^high^DP cells exhibiting Vβ5 and Vβ12 segments. However, despite higher numbers of immature thymocytes bearing such Vβ segments were noticed in the thymus, they were no longer detected in the intrathymic SP stage, implying that negative selection apparently occurs. Similar data were reported by other groups (110, 111). These results also indicate that DP thymocytes bearing prohibited Vβ12 segments escape from the thymus and gain the secondary lymph organs, where they further may differentiate into mature and potentially autoreactive CD4^+^ or CD8^+^ T cells (106). Supporting this idea, increasing numbers of CD8^+^ SP T cells exhibiting Vβ5 and Vβ12 segments were detected in the lymph nodes of BALB/c chronically infected mice (106).

Further studies showed that intrathymic key elements involved in the promotion of negative selection remain functional during the acute chagasic thymic atrophy (46). Moreover, using a transgenic system consisting in chicken egg ovalbumin (OVA)-specific T-cell receptor, it was shown that OVA administration in infected mice with thymic atrophy promoted OVA-specific thymocyte apoptosis, reinforcing the idea that a rather normal process of negative selection takes place during the infection (46).

Despite the fact that human beings have no TCR repertoire deletions compared with mice, during human Chagas disease there is also a differential expression of TCR-Vβ5 T cells from infected patients in different stages of the disease, and preferential expansion of CD4^+^Vβ5^+^ and CD8^+^ Vβ5^+^ T cells was also observed when mononuclear cells were exposed *in vitro* to parasite antigens, suggesting a possible commitment of these cells in the pathology (113). The preferential expansion of Vβ5^+^ populations occurs in both CD28^+^ and CD28^−^ CD4^+^ T cell subsets and is related to restricted sequences of CDR3 (114, 115). Interestingly, C57BL/6 mice, which do not suffer the clonal deletion of TCR-Vβ cell subsets, also expand peripherally CD8^+^ Vβ5^+^ clones during infection (112). Secondly, it was reported an increased frequency of T cells bearing Vβ7 in chronic and symptomatic patients bearing HLA haplotypes that were previously associated with susceptibility to cardiac damage (116). These data suggest that dominant antigenic epitopes may be involved in the expansion of peripheral T cells expressing specific Vβ regions. In this regard, some authors raised the bet proposing the existence of a *T. cruzi*-derived exogenous T superantigen-like moieties that may induce the development of a CD8^+^ Vβ-skewed response (112). Nevertheless, we cannot rule out that the escape of immature and potentially autoreactive T cells might shape the peripheral T cell repertoire. Reinforcing this hypothesis, we showed the presence of DP and DN cells bearing an activated phenotype in patients with chronic Chagas disease (46).

Additionally, it seems evident that the loss of tTreg cells may compromise the reposition of the Treg-peripheral pool. Such sustained alteration overtime may be partially related to the T-cell autoimmune events occurring in CCC (117, 118). It is interesting to note in this regard that even though Treg cells can develop “*de novo”* from CD4^+^ SP T cells in the periphery (pTreg cells), it have also been found an enhanced proportions of Treg cells in chronic but symptomless chagasic patients compared to those exhibiting CCC (117, 119, 120). Likely, in patients with CCC, Treg cells are not sufficient and competent to control the systemic inflammatory process and the cardiac lesions mediated by activated CD8^+^HLA-DR^+^ T cells and potentially autoimmune DP HLA-DR^+^ T cells. Unfortunately, there are no studies evaluating the tTreg and pTreg repertoire in mice and humans infected with *T. cruzi*. These studies may help to elucidate the nature of the rare self-antigens that induce Treg cell differentiation.

In addition, recent work showed that Th1 systemic response against *T. cruzi* is able to influence the intrathymic development of a particular population among CD8^+^ SP thymocytes: the “CD8^+^ innate cells or innate memory–phenotype” (T_IM_-CD8^+^) cells (43). As mentioned in Box 1, these subpopulation is generated as an independent lineage different from conventional CD8^+^ T cells (25, 26, 121). However, little is known concerning their intrathymic development in normal steady-state conditions and even less after pathological situations. Nevertheless, new evidence showed that enrichment of CD8^+^ SP thymocytes with an “innate phenotype” could be seen after Th1-driven infectious processes where systemic expression of IL-12 and IL-18 is triggered. This effect was particularly observed after infection with 2 different strains of *T. cruzi*. Of note, the systemic production of these inflammatory cytokines induced high expression of both IL-4 and IL-15 in the thymus, generating an appropriate niche for the conversion of DP thymocytes (an also potentially autoimmune DP cells) toward the innate phenotype over the conventional CD8^+^ SP pathway (43).

Taken together, these findings suggest that the release of potentially autoreactive T cells in the periphery of the immune system (Table 2), linked to potential abnormalities in the negative central tolerance mechanisms, might contribute to the autoimmune process found in both murine and human Chagas disease.

**Table 2:** Characteristics of extrathymic CD4^+^CD8^+^ double positive (DP), CD4^−^CD8^−^ double negative (DN) T cells and peripheral *TCR-V*β T cell repertoire in mice and humans infected with *T. cruzi*.

**Characteristics**	**Mice**	**References**	**Humans**	**References**
**CD4**^**+**^**CD8**^**+**^ **DP T cells**				
Extrathymic appearance during the acute phase	✓	(13, 46, 50, 106, 122)	ND	
Extrathymic appearance during the chronic phase	✓	(46)	✓	(46, 123)
Expression of activation markers	✓	(46)	✓	(46, 123)
Pro-inflammatory cytokine production	✓	(46)	✓	(123)
Enhanced expression of VLAs and chemokine receptors	✓	(50)	ND	
Granzyme/Perforin expression	✓	(46)	✓	(123)
Expression of prohibited segments TCR-Vβ	✓	(50, 106)	ND	
Presence in cardiac tissue	✓	(122)	✓	(123)
**CD4**^**+**^**CD8**^**+**^ **DN T cells**				
Extrathymic appearance during the acute phase	✓	(124, 125)	ND	
Extrathymic appearance during the chronic phase	✓	(106)	✓	(124–127)
Expression of activation markers	✓	(124, 125)	✓	(126)
Pro-inflammatory cytokine production	✓	(124)	✓	(126)
**Peripheral T- cell repertoire**				
Skewed TCR-Vβ expression in peripheral T cells	✓	(106)	✓	(113–116)

*ND, not determined*.

## Abnormal Intrathymic T-Cell Migration, Exit, and Re-Entrance Following Acute *T. cruzi* Infection

**Figure 2:**
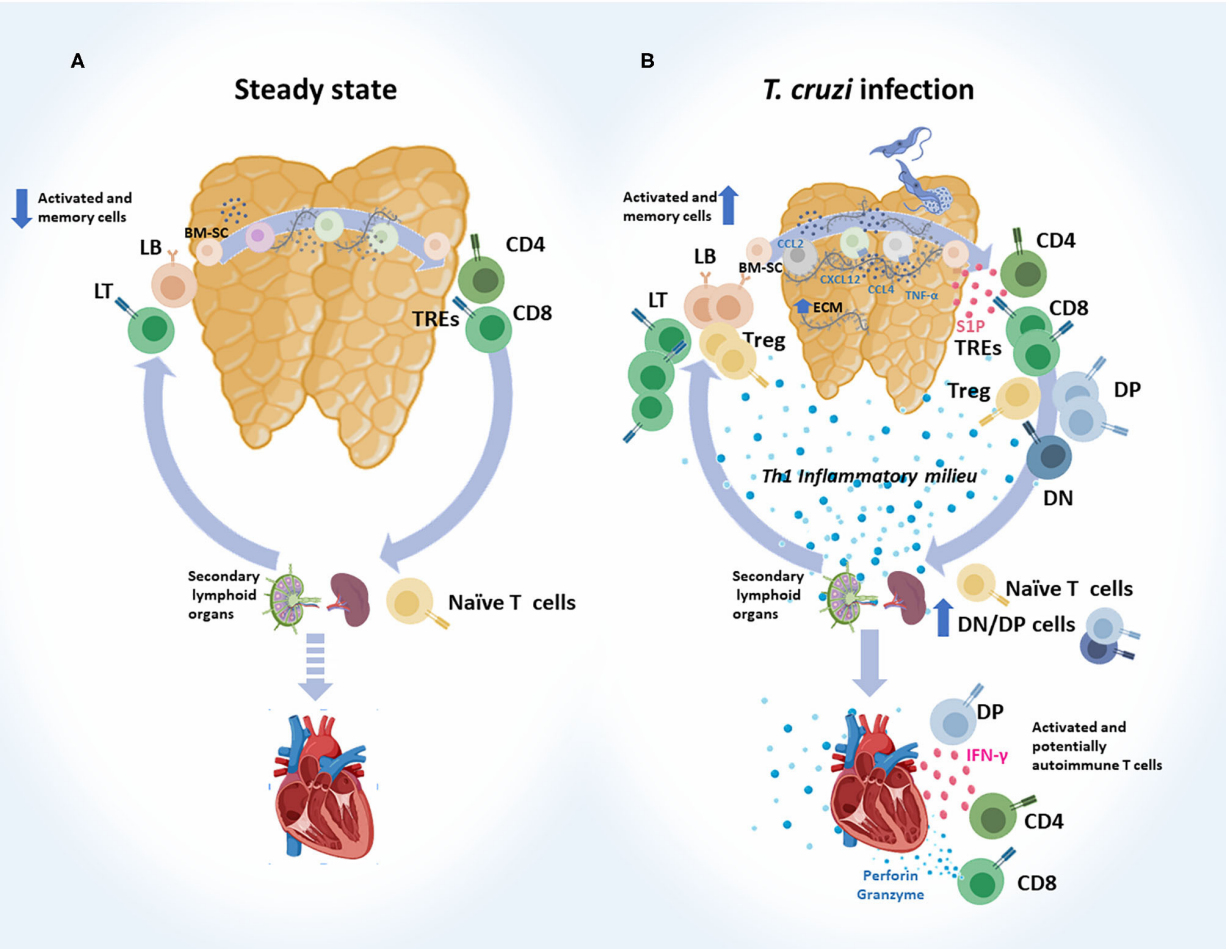
Abnormal intrathymic T-cell migration, exit and re-entry following acute *T. cruzi* infection. **(A)** Steady state. Normally, after bone marrow-derived stem cells (BM-SC) enter into the thymus and commit to the T cell lineage, thymocytes engage a complex journey through the organ. Stromal-derived signals like chemokines and cytokines, and also the interaction with extracellular (ECM) proteins, guide the traffic and maturation of developing thymocytes (DN → DP → SP). Selected thymocytes leave the organ as recent thymic emigrants (RTEs), mainly represented by mature CD4^+^ and CD8^+^ SP T cells. These cells migrate to secondary lymphoid organs and turn into naïve T cells. When encountering their specific antigen, naïve T cells become activated and migrate to target tissues to execute their effector functions. Some of them become memory T cells, and a very low proportion of memory/activated T cells may re-enter the thymus. **(B)** *T. cruzi* infection. Migratory properties of thymocytes and thymus-derived T cells are profoundly affected during murine acute *T. cruzi* infection. Intrathymically, these alterations are linked to enhanced ECM deposition, increased levels of CCL2, CXCR4, CXCL2, and TNF-α, which have haptotactic functions. DP thymocytes and tTregs are among the most affected cell types. Once the intrathymic development process is completed, cells leave the thymus, as recent thymic emigrants RTEs guided by an enhanced S1P gradient in the bloodstream. Conjointly with CD4^+^ and CD8^+^ SP cells, progressively increased proportions of DN and DP T cells escape from the organ, probably as a consequence of thymic microenvironmental abnormalities, together with intrathymic and systemic Th1 pro-inflammatory contexts. In mice, a proportion of SP and DP cells carried prohibited TCR-Vβ fragments. Naïve T cells may encounter *T. cruzi*-derived parasites in secondary lymph nodes and become effector T cells. Potentially, autoimmune DN and DP T cells detected in the periphery also showed an effector-like T phenotype. Effector T cells, target heart to control parasites and at the same time diverse autoimmune events may occur. In addition, increased proportion of memory/activated T cells re-enter the thymus. DN, double negative; DP, double positive; SP, single positive.

Cumulated evidence clearly shows that the intrathymic cell migration and both the exit and re-entry of T cells during infection are altered. These points are discussed in more depth below and schematically summarized in Figure 2.

### Intrathymic Migratory Response to Various Cell-Migration Related Molecules

**Figure 3:**
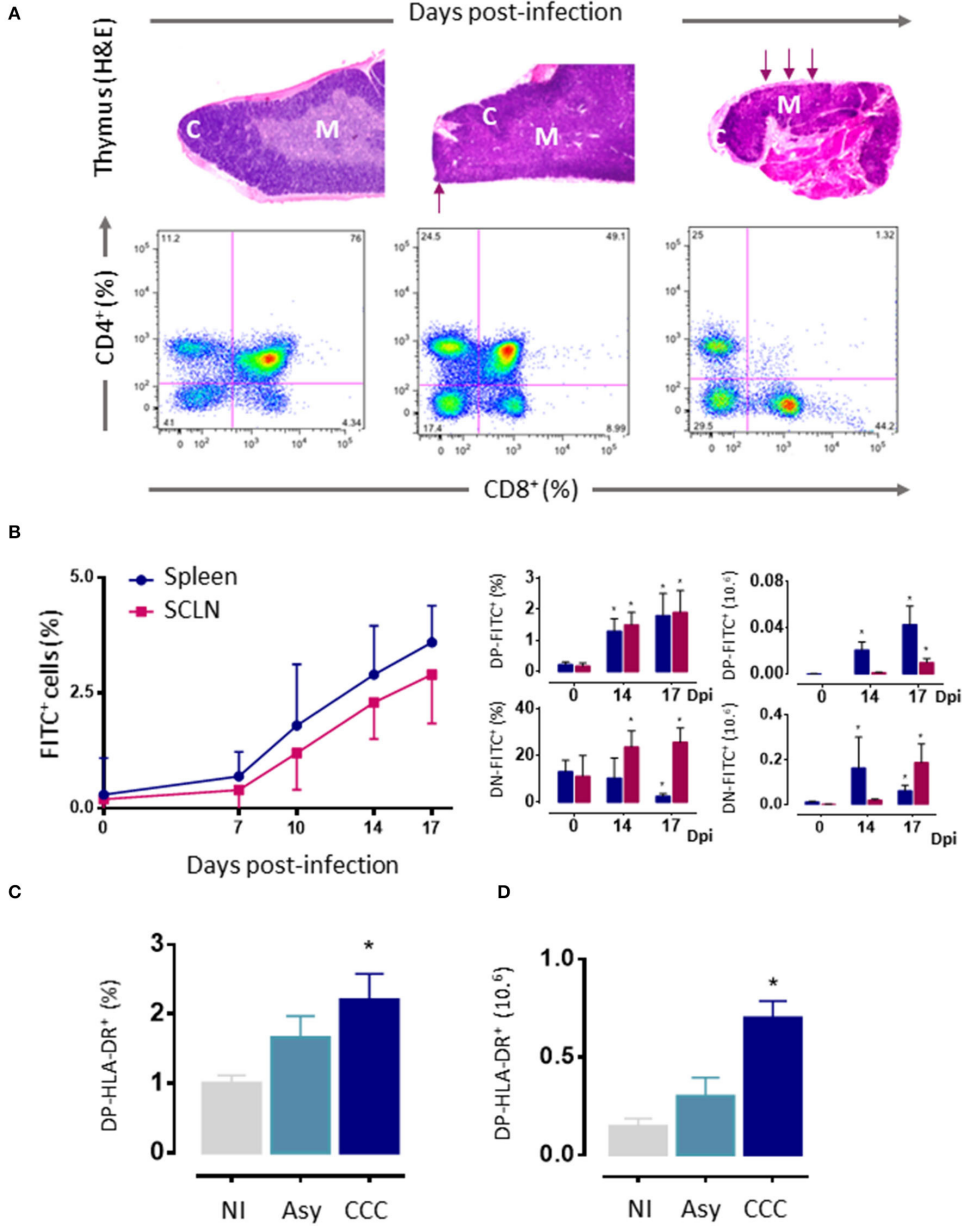
Thymic atrophy and abnormal exit of DP cells *T. cruzi* acutely infected mice. **(A)** Upper panels. Photomicrographs showing the progressive loss of cortical DP thymocytes during *T. cruzi* infection (H&E staining, 20X). Arrows indicate zones with cortical loss. Bottom panels: Representative dot plots showing an intense loss of the CD4^+^CD8^+^ DP subset, assayed by cytofluorometry. **(B)** Increased export of lymphocytes from the thymus of *T. cruzi* infected C57BL/6 mice. Acutely infected mice received intrathymic injection of FITC and were analyzed 16 h later by flow cytometry to detect recent thymic emigrants (FITC^+^ cells) in peripheral lymphoid organs. Left graph shows how the absolute numbers of FITC^+^RTEs progressively increased with the infection. Such increase comprises the abnormal release of immature CD4^+^CD8^+^ DP and CD4^−^CD8^−^ DN lymphocytes, whose relative and absolute numbers are progressively higher, in both subcutaneous lymph nodes and spleen (right graphs). **(C)** Circulating activated HLA-DR^+^DP cells in patients with chronic Chagas disease. DN, double negative; DP, double positive; RTEs, Recent thymic emigrants; NI, non-infected patients; Asy, asymptomatic patients without cardiopathy demonstrated; CCC, patients with chronic chagasic cardiomyopathy; H&E hematoxylin-eosin. **(A)** Was adapted from Villa-Verde et al. (79), **(B)** from Barbosa-ferreira et al. (7); Mantuano-Barradas et al. (81), and **(C)** from Acha-Orbea et al. (107). *p < 0.05.

As mentioned above, thymic atrophy and DP T cells loss caused by *T. cruzi* infection in mice (Figure 3A) are accompanied by thymic microenvironmental changes that include altered expression of migration-related components. These changes alter thymocyte intrathymic migration possibly influencing their development and inducing alterations in the export of thymocytes. One would suspect that changes in the expression of migratory-related proteins could lead to disturbances in the thymocyte journey throughout the thymic lobule. In fact, the migratory properties of thymocytes are profoundly affected during murine *T. cruzi* infection (13, 46, 50, 51, 128–130).

A broad diversity of chemokines is constitutively expressed in defined areas inside the thymic lobules, preferentially attracting a given particular subpopulation of thymocytes expressing the corresponding receptors. In particular, chemokines such as CXCL12 and CCL4 have major roles in the intrathymic migration of developing thymocytes and thymocyte precursors. CXCL12 is produced by TEC, mainly in the subcapsular, and medullary regions and mediates the initial migratory phases attracting DN cells from the cortico-medullary junction to the subcapsular zone, where specific signals induce and regulate the earliest stages of thymocyte development. Moreover, CXCL12 is involved in the migration of DP cells and the corresponding receptor CXCR4 is highly expressed in this population (131). Interestingly, marked migration disturbances concerning this chemokine were demonstrated in the thymus during experimental infection with both *T. cruzi* strains. Thymuses from infected animals showed a CXCL12 distribution denser and not organized, likely due to the virtual disappearance of the cortical region, while the remaining DP cells increased the expression of the corresponding receptor CXCR4 (51). These findings were paralleled by an upregulation of CCL4 expression and an increased density of the respective receptor CCR5 on DP cells. A further aspect to be discussed is that CXCL12 binds and it is also presented by ECM proteins like fibronectin. In this regard, enhanced CXCL12 and fibronectin co-localization was clearly detected in thymuses from *T. cruzi* infected animals. Additionally, *ex-vivo* experiments showed that fibronectin/CXCL12 enhanced the haptotactic migration of immature thymocytes from *T. cruzi* infected mice as compared to controls, suggesting that fibronectin increases CXCL12 sequestration, thus facilitating its presentation to thymocytes and enhancing their migration toward this chemokine (51).

The ECM-driven migration is settled mainly by the interaction of thymocytes with TEC. A progressive increase in the expression of fibronectin and laminin, in both cortex and medulla of thymic lobules, was observed in *T. cruzi* infected mice. When cortical thymic nurse cells were exposed *in vitro* or *in vivo* to *T. cruzi* there was an increase in the deposition of ECM proteins, with an intensified release of DP cells from the lymphoepithelial complexes (23). In parallel, an enhancement in the expression of corresponding very late antigen(VLA)-4, VLA-5, and VLA-6 receptors on thymocytes was observed in the thymus of *T. cruzi* infected mice (42, 50).

In a second vein, the expression of TNF-α is also enhanced in the thymus during *T. cruzi* infection and *ex-vivo* studies suggest that, when TNF-α is complexed with fibronectin, it favors the migratory capacity of DP thymocytes derived from *T. cruzi* infected animals (129). Besides cytokines and chemokines, ECM components might adsorb parasite-derived antigens, contributing to the establishment and perpetuation of *in situ* reactions. In this respect, it is noteworthy that the parasite antigen cruzipain binds fibronectin in myocardial tissue possibly favoring T cell infiltration while trans-sialidase promotes fibronectin-driven thymocyte migration (130, 132).

The intrathymic journey of thymic regulatory CD4^+^Foxp3^+^ Treg cells also seems to be affected during *T. cruzi* infection. In addition to studies showing a marked loss of tTreg cells in the thymus of infected mice, an abnormal cortical localization of Foxp3^+^ cells were detected. Strikingly, the membrane expression levels of the chemokine receptors CCR7 and CXCR4 are enhanced in this population. Notoriously, in normal and infected thymus, the Foxp3^+^ population expressed a higher proportion of both receptors compared to Foxp3^−^ cells, but the membrane level of both receptors (ascertained by MFI) was diminished in Foxp3^+^ cells from infected animals. Moreover, fibronectin-driven migration of Foxp3^+^ thymocytes from infected animals was clearly diminished in *transwell* cell migration assays, associated to a diminution in VLA-4 and VLA-5 (90). These findings support the notion that tTreg cells may undergo alterations in their trafficking capacity during *T. cruzi* infection, although functional studies in this particular cell subset remain to be done.

Overall, alterations in the intrathymic expression of ECM proteins, soluble chemoattractant molecules, and cytokines, as well as changes in the levels of integrins and chemokine receptors, likely contribute to thymocyte migratory alterations observed during *T. cruzi* infection.

### Alterations in Thymic Exit and Extrathymic Immature T Cells

Changes in the intrathymic migration during *T. cruzi* infection are linked to the abnormal appearance of thymus-derived immature DN and DP T lymphocytes in peripheral lymphoid organs and blood from infected hosts (13, 50, 124); features that are summarized in Table 2. Accordingly, this premature leakage in thymocyte developmental terms of immature cells from the thymus may also contribute to the establishment of the thymic atrophy observed during parasite infection.

As already commented herein, experimental *T. cruzi* acute infection induces a strong polyclonal T-cell activation with an over-representation of some TCR-Vβ families (112), which overlap the presence of extrathymic immature DP T cells expressing potentially autoreactive TCRs (Table 2) (50, 106, 133). Although this phenomenon may be a consequence of failure in the negative selection process, the data available consistently indicate that thymopoiesis (including negative selection events) remains functional during the *T. cruzi* infection (46), reinforcing the notion that the abnormal release of immature T lymphocytes is rather related to intrathymic migratory disturbances. In normal conditions, the signaling pathway mediated by the bioactive lipid sphingosine-1 phosphate (S1P) through its receptors (S1PRs) is responsible for the exit of mature SP cells from the thymus (46). In this regard, it was demonstrated that thymic S1P availability plays a role in the exit of immature DN cells in experimental Chagas disease (125) and probably in the escape of DP cells. At the thymic level, both S1P and the balance of enzymes involved in the S1P pathway during *T. cruzi* infection are altered, showing a reduction of S1P paralleled by a downregulated gene expression of the kinase SPHK1/2 (the enzyme that forms S1P by the phosphorylation of sphingosine) and up-regulation of the phosphatase SGPL1 (the enzyme involved in the terminal breakdown of S1P)(125). Since S1P levels are not altered in the serum of infected animals, the S1P gradient (S1P in thymus < S1P serum) that is established favors the exit of the cells from the thymus. Moreover, increased membrane expression of S1PRs (particularly S1P1 and S1P3) on DN and DP thymocytes was observed following *T. cruzi* infection (125). The blockade of S1P receptors with FTY720 avoided the escape of DN to the periphery and consequently restored physiological proportions of DN thymocytes in infected mice (125), demonstrating that S1P-mediated pathways contribute to the premature exit of these cells.

In humans, extrathymic DP T cells have been described in pathological conditions such as diverse infectious diseases, autoimmune diseases, chronic inflammatory disorders, and certain lymphoblastic diseases (134–136). In *T. cruzi* infected mice, the first evidence of an aberrant thymic release of DP lymphocytes to the periphery was the observation that these cells progressively accumulated in spleen and subcutaneous lymph nodes (Figure 3B) (13). Some studies support the idea that such DP cells may be memory CD4^+^ helper T cells that, after activation, have acquired the ability to retain the expression of the CD8α chain (123, 135, 137). Nevertheless, in our studies, thymocytes were previously intrathymically labeled with a fluorescent dye and 16–24 h later tracked in peripheral lymphoid organs (13), precluding the possibility that these cells can be quickly differentiated into memory cells. Actually, DP T cells can also be detected in the circulation and, in low numbers, within the heart of acutely infected mice (122). The augmented presence of these cells in peripheral organs (including the heart) may represent an accelerated recruitment of T cells from thymus as a compensatory mechanism to overcome the anergy/immunosuppression described during the acute phase of *T. cruzi* infection (138). Accordingly, these peripheral DP cells exhibit high densities of ECM and chemokine receptors (50) and an activated phenotype, showing increased IFN-γ production and cytotoxic activity (107). These data are strongly related to humans, with the presence of activated HLA-DR^high^ DP T cells in patients with the cardiac form of the disease (Figure 3C). Such an increase, not seen in patients in the asymptomatic form, suggests a positive correlation between the frequencies of circulating and tissue DP cells and the presence of cardiomyopathy (46, 123), by far the most important clinical consequence of *T. cruzi* infection (46, 123). Yet, the low proportion of these cells in the blood of patients makes it difficult to isolate them in a sufficient quantity for functional studies and, therefore, also hinders their clinical implication. In addition, in the mouse model, similar results were observed with peripheral DN T cells, which exhibit increased mRNA levels for TNF-α and IL-17A upon polyclonal activation (124). Moreover, αβ and γδ DN T cells have been also detected in the blood of patients with chronic Chagas disease (126, 127). Interestingly, while αβ DN T cells from CCC patients expanded *in vitro* showing a clear pro-inflammatory profile, γδ DN T cells from indeterminate patients exhibited an anti-inflammatory profile (127). Overall, these findings suggest that DN T cells may be linked with the establishment of different clinical forms of Chagas disease.

Despite the mechanisms underlying the premature thymocyte release during the chagasic thymic atrophy remains elusive, some data suggest that parasite-derived antigens could be involved in the altered exit of immature T cells since intrathymic injection of trans-sialidase in mice enhances the abnormal release of DP cells (130). Hypothetically, trans-sialidase might also play a role in the altered release of DP cells in patients with the indeterminate or cardiac clinical forms of Chagas disease; since there was a gradual enhancement of antibody titres against trans-sialidase as the frequency of the peripheral blood DP cell subset increases (130).

In addition, abnormal thymic exit can also be associated with the immunoendocrine imbalance that takes place during *T. cruzi* infection in both mice and humans (122). In patients with chronic Chagas disease, extrathymic DP T cells positively correlated with circulating levels of TNF-α and with the cortisol/DHEAS ratio, particularly in patients having CCC (122). This raises the question of whether there is a cause/effect relationship between the immunoendocrine alterations and the proportion of extrathymic DP T cells linked to clinical progression in humans with Chagas disease. In these individuals, the establishment of a chronic inflammatory milieu may affect the activated state of DP T cells, and at the same time, the cortisol/DHEAS ratio imbalance may act as a permissive scenario to myocarditis development. In this respect, further investigation on the relationship among TNF-α, DHEAS, and the cortisol/DHEAS ratio with the phenotype and effector functions of extrathymic DP cells, may help to elucidate the contribution of these cells to the progression to CCC (122).

A further issue that remains to be explained is whether or not differences seen in the regional immunology following *T. cruzi* infection are somehow related to the exit of T cells from the thymus. It has been shown that splenomegaly and lymphadenopathies, particularly the expansion of subcutaneous lymph nodes is observed in experimental models and patients, mainly secondary to T and B cell polyclonal activation (139–142). Conversely, mesenteric lymph nodes (MLN) and Peyer's Patches are described to be largely reduced in experimental models of acute infection (42, 52, 143–145). Splenomegaly and subcutaneous lymph node hypertrophy are consequences of tissue T/B lymphocyte activation and expansion (139, 141, 146). In contrast, the MLN atrophy revealed a reduction of T and B lymphocyte numbers, as well as a decrease in IL-2, IL-4, and IL-10 production by activated T cells (54). The mechanism involved in IL-2 deprivation is this particular group of lymph nodes is not clear but can be associated to the differential distribution of Tregs, since IL-2 could be produced in normal levels and be sequestered by these cells in secondary lymphoid organs during infection (54, 147). Additionally, MLN cells from infected mice show reduced capacity to proliferate and enhanced apoptosis mediated by Fas, TNFR1/p55 and IL-4 deprivation, through caspase 9 activation (70, 148). Noticeably, apoptosis is also elevated in Peyer's Patches during acute infection (54). Overall, it seems plausible to raise the hypothesis of a differential homing of Tregs from the thymus to the periphery; such difference being related to the expansion vs. a contraction of T and B cells in peripheral lymphoid organs. Although difficult to be settled (due to the small amounts of Tregs in the thymus), such a possibility can be experimentally tested by studying RTEs, comparing the numbers of recent thymic Treg emigrants in various lymphoid organs.

### Re-entry of Mature T Cells Into the Thymus of *T. cruzi* Infected Mice

Under physiological conditions, the re-entry of mature T cells into the thymus is mainly restricted to peripheral cells that have acquired an activated phenotype or also memory cells. Yet, a non-negligible proportion of cells that recirculate back to the organ exhibit a naïve phenotype (149–151). This process has been described during the early phase of a Th1 inflammatory/infectious process. In this scenario, the cellularity of the thymus is seriously compromised, a situation that fits well with murine *T. cruzi* infection. Actually, it was demonstrated that a large number of mature peripheral B and T cells could enter the thymus in *T. cruzi*-infected mice, when atrophy is evident, compared to the number observed in non-infected mice where the thymus is intact (152).

The reasons for cell migration of both T and B cells back to the thymus have been addressed by several groups. In the case of peripheral B cells, it has been postulated their entry in the thymus would allow T-cell tolerance to immunoglobulins and to other B-cell-specific antigens (153). Others have proposed that B cells found in the thymus could participate in negative selection by acting as antigen-presenting cells (154). As for T cells, it has been suggested that the thymus can function as a repository of memory T cells (155), while others have demonstrated an important role of peripheral mature T cells in maintaining medullary epithelial cells (MEC) (156). The authors provided evidence indicating that i.v. injected T cells from lymph nodes are able to localize in the medullary region of the murine thymus and led to marked regeneration of MEC (156). Yet, such results need more undoubtful confirmation.

Furthermore, in cases of infection, the thymus might recruit immune cells specific to the invading pathogens that could ultimately lead to microbe-specific tolerance, impairing host resistance. This effect has been reported for *Mycobacterium avium* (157, 158), *Murine leukemia virus* (MLV) (159), *Lymphocytic choriomeningitis virus (LCMV)* (160) and *Hepatitis virus* (HBV) infections (161). Regarding to viral infections, it is possible that the intrathymic presence of viral proteins was responsible for inducing pathogen-specific T cell tolerance since both MLV and HBV, as well as Zika virus, can infect TECs (159, 162). As for *T. cruzi* infection, our recent work demonstrated both the presence of the parasite inside murine thymic macrophages and epithelial cells, along with a large number of activated/memory CD8^+^ CD44^high^ cells specific for one of the most immunogenic parasite antigen, the antigen TSKB20, a trans-sialidase derived epitope (43) However, new data are needed to demonstrate if these events can trigger parasite-specific tolerance as well.

Despite several laboratories have described the phenomenon of mature peripheral cells migrating into the thymus, there is a lack of evidence that determines the mechanism by which this process occurs. Nonetheless, it has been demonstrated that T cells able to enter the thymus of *T. cruzi*-infected animals exhibit enhanced expression of CD44 and CD62L but low expression of CD69, compatible with a central memory phenothype. Interestingly, blocking CD62L with neutralizing antibodies did not affect the immigration of mature T or B into the thymus, indicating that the process is independent of this selectin (152). In a second vein, in the infected organ, the enhanced expression of CCL2 is accompanied by an increase in the number of CCR2^+^ T cells. Accordingly, the administration of irbesartan (antagonist of CCL2) diminished the entrance of T cells, suggesting that the thymic increase in the contents of the chemokine facilitates the recruitment of peripheral CCR2^+^ lymphocytes (152).

It should be noted, however, that the ability of peripheral T cells to migrate into the thymus does not seem to be restricted to those cells bearing the activated/memory phenotype. This notion is reinforced by findings showing intrathymic tTreg cells with a marked maturational profile (90), suggesting that at least, a proportion of tTreg detected in the thymus may correspond to pTreg cells that have re-entered the organ. Interestingly, the appearance of these cells is detected when the atrophy is well-established, as Hodge and colleagues proposed earlier (152). Although the functional relevance of Treg cell re-entry into the thymus of during *T. cruzi* infection is still unknown, it is possible that peripheral Tregs and T effector cells that regain the organ regulate intrathymic tTreg differentiation. In this regard, some authors suggest that the re-entry of activated pTregs cells bearing the phenotype CD62L^−^CXCR4^+^ into the thymus might inhibit IL-2-dependent differentiation of tTreg cells (151, 163). Others speculate that the re-entry of T effector cells may contribute to the induction of tolerance by promoting tTreg development, by acting as a source of IL-2 (164). Whatever the case, remaining tTregs observed during *T. cruzi* infection express increased and diminished levels of CD62L and CD184/CXCR4, respectively (149).

## Concluding Remarks and Perspectives

**Figure 4:**
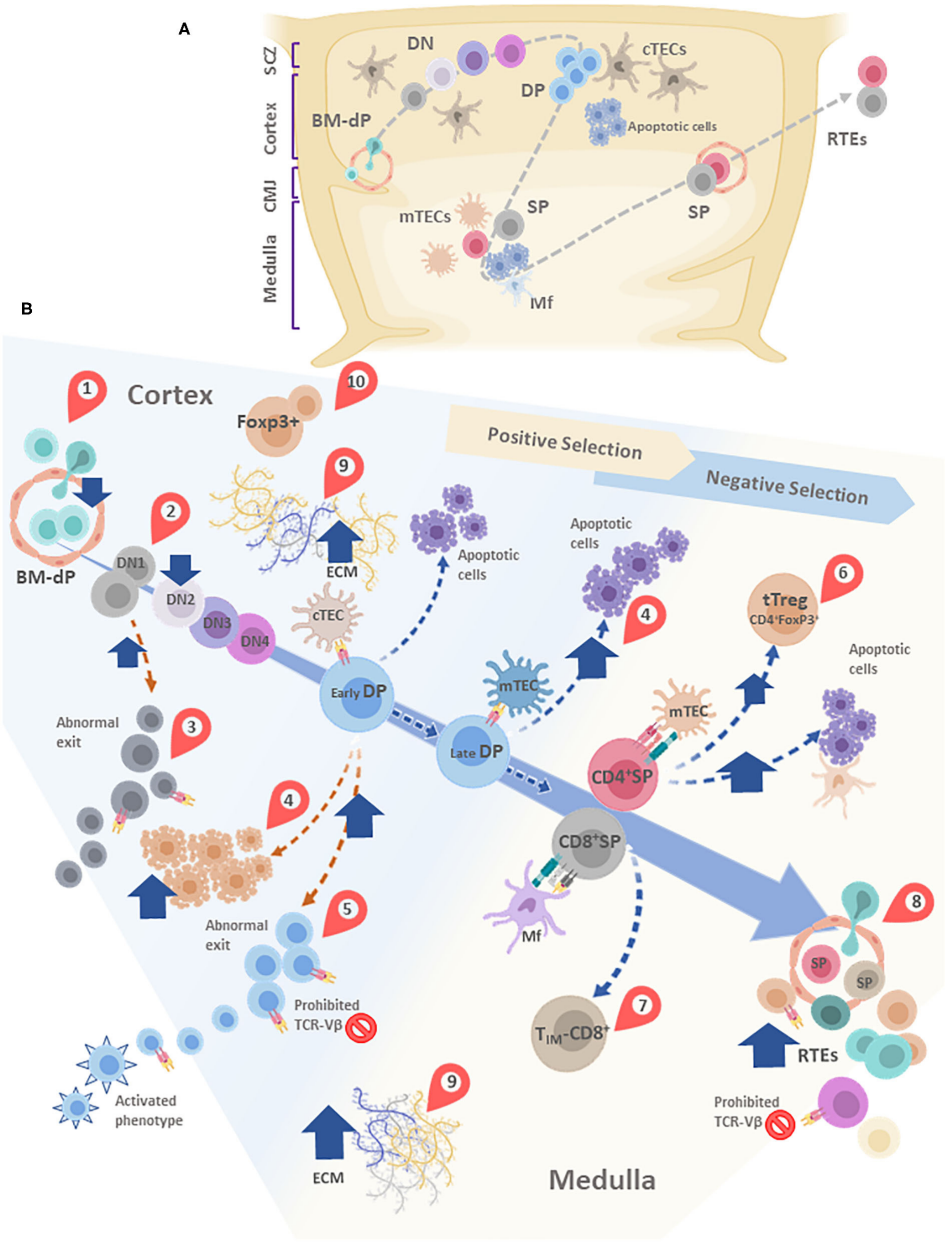
**(A)** Simplified schematic model of thymocyte migration across the thymic lobule. **(B)** Conveyor belt representation of thymocyte maturation and the processes of positive and negative selection. BM-derived precursors enter through blood vessels located in the cortico-medullary junction (CMJ). The most immature thymocytes originated from these pluripotent precursors are called DN cells, since lack CD4 and CD8. DN cells go through four stages (DN1-DN4) defined by the expression of CD44 and CD25. During differentiation, DN cells migrate from the CMJ to the subcapsular zone of the thymus. Cell precursors are irreversibly committed to the T-cell lineage during the passage from DN1 to DN2 state. In this stage, cells that are successful in the rearrangement of the β-chain of the T-cell receptor (TCR) are selected for further maturation steps. After passing by the DN4 stage, survived thymocytes are converted into double-positive (DP) thymocytes by acquisition of CD4 and CD8. In this step occurs the rearrangement of the genes encoding the α-chain of the TCR. Double-positive thymocytes undergo are positive and negative selection, and their destinies are determinated by the interaction with the thymic epithelial cells (TECs). Once positively selected, tjey are able to generate single-positive (SP) cells. In this step, thymocytes move away from de cortex and enter the medulla. A large percentage of thymocytes die by neglect, due to the non-functionality of their TCRs. The remaining cells interact with different degrees of affinity with their specific antigens. A strong affinity prompts apoptosis and subsequent clonal deletion. An intermediate affinity may induce regulatory T cells. Also, T_IM_-CD8: Innate memory CD8 cells appear. Low affinities allow their differentiation to SP cells. Finally, mature thymocytes reach the CMJ and exit to the periphery as RTEs. In most of these stages, diverse alterations are observed after *T. cruzi* infection, which are indicated in the figure by red teardrop pointers: (1) Decreased entrance of BM-dP; (2) Retention of thymocytes in the DN1 state; (3) DN abnormal escape; (4) Enhanced DP thymocyte apoptosis, mainly driven by enhanced levels of glucocorticoids. (5) DP abnormal escape, with many of them carrying prohibited TCR-Vβ, also exhibiting an activated phenotype; (6) tTreg accumulation among SP thymocytes; (7) Increase in T_IM_-CD8 cells; (8) Increase in RTE proportions, with many of them carrying prohibited TCR-Vβ; (9) Increased deposition of ECM molecules in both cortex and medulla, (10) Abnormal presence of FoxP3^+^ cells in the cortical area. cTEC, cortical thymic epithelial cells; mTEC, medullary thymic epithelial cells; DC, dendritic cell; Mf, macrophages; DN, double negative; DP, Double positive; SP, single positive; BM-dP, Bone marrow-derived precursors; CMJ, Corticomedullary junction; RTEs, Recent thymic emigrants; T_IM_-CD8, Innate memory CD8^+^ cells; SCZ, Subcapsular Zone (SCZ); ECM, extracellular matrix.

The most important concept emerging from the data discussed above is that *T. cruzi* infection disrupts intrathymic homeostasis, in terms of both microenvironmental and lymphoid compartments of the organ. As summarized in Figure 4, almost all stages of thymocyte development are altered: caspases-dependent massive apoptosis, changes in the amounts of tTreg cells, qualitative changes in the intrathymically repertoire, altered expression of cell-migration related receptors, together with an abnormal exit of immature DN and DP T cells. Whether all these changes occur in humans is to be determined, although at least abnormal DP and DN lymphocytes have been found in patients with Chagas disease.

The further knowledge on the molecular mechanisms underlying thymic abnormalities occurring during *T. cruzi* infection, as well as the consequences of the thymic abnormalities upon the peripheral immune response to the parasite, may contribute to designing innovative strategies to control Chagas disease pathology.

## Author Contributions

All authors listed have made a substantial, direct and intellectual contribution to the work, and approved it for publication. AP made figures.

## Conflict of Interest

The authors declare that the research was conducted in the absence of any commercial or financial relationships that could be construed as a potential conflict of interest.
